# Development and evaluation of a real-time one step Reverse-Transcriptase PCR for quantitation of Chandipura Virus

**DOI:** 10.1186/1471-2334-8-168

**Published:** 2008-12-17

**Authors:** Satyendra Kumar, Ramesh S Jadi, Sudeep B Anakkathil, Babasaheb V Tandale, Akhilesh Chandra Mishra, Vidya A Arankalle

**Affiliations:** 1 National Institute of Virology, 130/1, Sus Road, Pashan, Pune, India, 411021

## Abstract

**Background:**

Chandipura virus (CHPV), a member of family *Rhabdoviridae *was attributed to an explosive outbreak of acute encephalitis in children in Andhra Pradesh, India in 2003 and a small outbreak among tribal children from Gujarat, Western India in 2004. The case-fatality rate ranged from 55–75%. Considering the rapid progression of the disease and high mortality, a highly sensitive method for quantifying CHPV RNA by real-time one step reverse transcriptase PCR (real-time one step RT-PCR) using TaqMan technology was developed for rapid diagnosis.

**Methods:**

Primers and probe for P gene were designed and used to standardize real-time one step RT-PCR assay for CHPV RNA quantitation. Standard RNA was prepared by PCR amplification, TA cloning and run off transcription. The optimized real-time one step RT-PCR assay was compared with the diagnostic nested RT-PCR and different virus isolation systems [*in vivo *(mice) *in ovo *(eggs), *in vitro *(Vero E6, PS, RD and Sand fly cell line)] for the detection of CHPV. Sensitivity and specificity of real-time one step RT-PCR assay was evaluated with diagnostic nested RT-PCR, which is considered as a gold standard.

**Results:**

Real-time one step RT-PCR was optimized using *in vitro *transcribed (IVT) RNA. Standard curve showed linear relationship for wide range of 10^2^-10^10 ^(r^2 ^= 0.99) with maximum Coefficient of variation (CV = 5.91%) for IVT RNA. The newly developed real-time RT-PCR was at par with nested RT-PCR in sensitivity and superior to cell lines and other living systems (embryonated eggs and infant mice) used for the isolation of the virus. Detection limit of real-time one step RT-PCR and nested RT-PCR was found to be 1.2 × 10^0 ^PFU/ml. RD cells, sand fly cells, infant mice, and embryonated eggs showed almost equal sensitivity (1.2 × 10^2 ^PFU/ml). Vero and PS cell-lines (1.2 × 10^3 ^PFU/ml) were least sensitive to CHPV infection. Specificity of the assay was found to be 100% when RNA from other viruses or healthy individual was used.

**Conclusion:**

On account of the high sensitivity, reproducibility and specificity, the assay can be used for the rapid detection and quantitation of CHPV RNA from clinical samples during epidemics and from endemic areas. The assay may also find application in screening of antiviral compounds, understanding of pathogenesis as well as evaluation of vaccine.

## Background

During an outbreak investigation of suspected Dengue and Chikungunya (CHIK) in Nagpur, Maharashtra state, India, Chandipura virus (CHPV) was isolated from 2 febrile cases and named after the locality from where the samples were collected [1]. CHPV belongs to the family *Rhabdoviridae*, genus *vesiculovirus *and is characterized by bullet shaped particles, 150–165 nm long, 50–60 nm wide [2].

CHPV is emerging as an important encephalitis-causing pathogen in India. Outbreaks of the disease have been reported among children from the states of Andhra Pradesh, Gujarat and Maharashtra [[2,3], National Institute of Virology (NIV) unpublished data] with mortality ranging from 55–75%. Death occurred within 48 hr of onset of symptoms and spread in the focal area rapidly. Considering the importance of the emerging pathogen, understanding the pathogenesis of CHPV infection in humans and experimental animals assumes immediate priority. For such studies as well as for the evaluation of potential candidate vaccine(s) and antiviral treatment strategies sensitive and specific viral quantitation assays are necessary. Earlier studies have shown the effectiveness of real-time PCR to detect and quantitate viral load, and have shown its utility as an indicator of the extent of active infection [4-6]. Currently, CHPV quantitation is done *in vivo *(mice), *in ovo *(Eggs) and *in vitro *(cell-lines) and virus titer is determined by 50% endpoint method [7]. However, these methods are time consuming and labor intensive and have inherent limitations in quantifying viral concentration in a large number of clinical samples. Another important issue with CHPV encephalitis is diagnosis. As the course of the disease is very rapid with high mortality, detection of viral RNA by nested RT-PCR represents the diagnostic method of choice rather than IgM-anti-CHPV antibodies [2,3].

Real-time one step RT-PCR assay offers an excellent alternative with several advantages such as high sensitivity, speed, accuracy and reproducibility [8]. The assay allows handling of a large number of samples in a short time and facilitates ways for automation [9]. Possibility of contamination associated with nested RT-PCR format can also be avoided. We therefore made an attempt to develop a highly sensitive real-time one step RT-PCR assay for CHPV and evaluated its potential with diagnostic nested RT-PCR and conventional means for virus detection. Efficacy of real-time one step RT-PCR was also compared with diagnostic nested RT-PCR, which is considered as a gold standard.

## Methods

### Virus and RNA isolation

The CHPV isolate used in this study was CIN0327R, isolated during the outbreak in Andhra Pradesh in 2003 [2]. Viral RNA was extracted from each sample using silicon-based spin columns (QI Amp viral RNA minikit, QIAGEN, Valencia, CA) as described by the manufacturer. 140 μl of clinical samples were taken for RNA isolation, samples having less than desired volume was adjusted to 140 μl with DNase-RNase free water and RNA was eluted into 40 μl elution buffer.

### Designing of Primers and Probes

**Figure 1:**
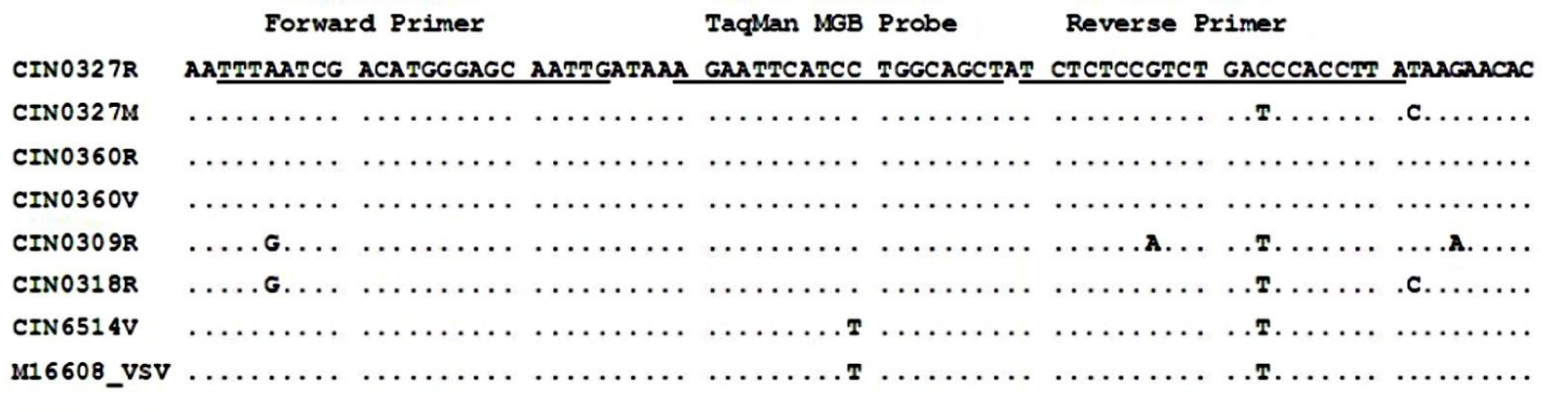
**Sequence alignment of different isolates of CHPV present in GenBank showing the location of primers and probe**. The primers and probe were located in CIN0327R (Accession no AY614726) from 1953–2022.

The CHPV P gene sequences available in the GenBank database were aligned using MEGA3 software [10]. The conserved regions were targeted to design primers and TaqMan Minor groove Binder (MGB) probes using the Primer Express software™ 2.0 (Applied Biosystems International, Foster City, CA) (Fig 1). Probe contained 5'-VIC reporter and 3' NFQ (Non fluorescent quencher) dyes. Applied Biosystems International, Foster City, CA, synthesized the primers and probes.

### RNA standard

A 591 bp fragment of P gene was amplified using NF5 and NR5 primers (Table 1). The PCR product was cloned between T7 RNA polymerase and SP6 polymerase promoter into pGEM T easy vector (Promega, Madison, USA). The plasmid with P gene was *in vitro *transcribed (IVT) by RiboMax™ Large Scale RNA production system T7 (Promega, Madison, USA) according to the manufacturer's instructions. To remove plasmid DNA, 40 units RNase-free DNase™ (Promega, Madison, USA) enzyme was used. Trizol LS reagent (Invitrogen, Carlsbad, CA) was used for RNA isolation according to manufacturer's instructions. The RNA concentrations were estimated by spectrophotometry based on the average of four measurements.

**Table 1:** Primers and Probe used in the study.

Assay	Primer/Probe	Sequence (5' → 3')	Position
P gene			
NF5	Forward Primer	TGAGTGCTCTCCAACTTCTGCAGT	1682–1705
NR5	Reverse Primer	TTCTTCAGAGCTTGCATCTTGAT	2331–2309
PF1	Forward Primer	TTTAATCGACATGGGAGCAATTG	1953–1975
PR1	Reverse Primer	TAAGGTGGGTCAGACGGAGAGA	2021–2000
PP	TaqMan MGB Probe	VIC-AGAATTCATCCTGGCAGCT-NFQ	1980–1998
1st PCR			
CHPNDGF3	Forward Primer	TGATTCCTACATGCCCTATCT	821–841
CHPNDGR3	Reverse Primer	GAACTTCTTCCCGTTAAGCACG	1078–1057
Nested 2nd PCR			
CHPNDGF4	Forward Primer	TCCACGAAGTCTCCTTACTCT	863–883
CHPNDGR4	Reverse Primer	GCACGAATCTCTGCTCCAGCT	1061–1041

The primers and probe were located in CIN0327R (Accession no genAY614726AY614726).

### Real-Time one step RT-PCR Assay

The real-time RT-PCR assay was performed using the real-time one step RT-PCR master mix (Applied Biosystems International, Foster City, CA). The real-time assays were done in 25 μl of reaction volume which contained 12.5 μl of 2× Master Mix, 0.6 μl of 40 × multiScribe RT and RNase inhibitor mix, 500 nM of PF1 forward primer and PP1 TaqMan MGB probe was used with 300 nM of PR1 reverse primer (Table 1). 5 μl of IVT RNA was used in each reaction. DNase-RNase free water was used to adjust the volume to 25 μl. Real-time one step RT-PCR was performed in a 96 well format using 7300 Real time PCR system (Applied Biosystems International, Foster City, CA) and SDS software version 1.3.1. The real-time one step RT-PCR employed the following thermal cycler settings: 30 min at 48°C, and 10 min at 95°C, followed by 50 cycles of 15 sec at 95°C and 1 min at 60°C. Each real-time one step RT-PCR assay had at least two no template controls (NTC) in which RNA was substituted by DNase-RNase free water.

### Reproducibility

Reproducibility of the assay was examined by running the standards on six different days in triplicate and threshold cycle (Ct) were recorded for different input copies of IVT RNA.

### Comparison of sensitivity of real-time one step RT-PCR with diagnostic nested RT-PCR and different conventional systems

For the comparison of different assay systems, CHPV of known titer (1.2 × 10^7 ^PFU/ml) was diluted serially (10-fold) in MEM containing 2% FBS. These dilutions were aliquoted and used for *in vitro*, *in vivo*, *in ovo *and PCR analyses. Irrespective of the volume of inoculum, all systems received equal number of virus particles. For real-time one step RT-PCR analysis and nested RT-PCR, viral RNA was extracted from each dilution as described earlier by using silicon-based spin columns (QI Amp viral RNA minikit, QIAGEN, Valencia, CA) followed by nested RT-PCR/real-time one step RT-PCR. Different systems were compared for CHPV detection limits.

#### (i) Cell lines

The vertebrate cell lines (Vero E6, PS and RD cell lines) used in the study were maintained in MEM supplemented with 10% FBS, while the sand fly cell line was maintained in Grace's insect cell culture medium supplemented with 15% FBS. FBS and culture media were procured from Gibco/Invitrogen, Corporation, Carlsbad, CA.

#### (ii) Infection of cell lines

The vertebrate and invertebrate cell lines were seeded in 96-well plates (Nunc, Denmark) and tissue culture tubes (TPP, Switzerland) respectively. The vertebrate cells were infected with 100 μl virus suspension at 80–90% confluency (n = 4) and incubated at 37°C. The insect cells grown on cover slips (9 × 22 mm) were infected with 100 μl virus suspension as above in quadruplicate and incubated for 1 hr at 28°C with intermittent rocking. After incubation, the inoculum was discarded, washed three times with sterile PBS, fed with Grace's insect cell culture medium supplemented with 10% FBS and incubated at 28°C.

#### (iii) Embryonated egg inoculation

Twelve-day-old embryonated special pathogen free (SPF) chicken eggs procured from Venkatershwara Hatcheries, Pune were inoculated with different dilutions (200 μl/egg) of CHPV (n = 4 per dilution) through the allantoic route and incubated at 37°C with 90% humidity and observed for 48 hr. The mortality was recorded by candling the eggs at different post infection (PI) hour. Eggs inoculated with normal saline were kept as negative controls.

#### (iv) Mice inoculation

Two-day-old infant Swiss albino mice were inoculated intra-cerebrally with different dilutions of CHPV (n = 8). Each group received 20 μl of virus inoculum. Control group was inoculated with normal saline. The mice were monitored for sickness and mortality.

### Sensitivity of different systems

#### (i) *In vitro *systems

The vertebrate cells were observed daily for cytopathic effects (CPE) under an inverted microscope and stained after 48 hr PI with amido black. Since the insect cells did not show CPE, indirect immunofluorescent antibody (IFA) technique was used to determine the presence of antigen in the cells infected with different dilutions of virus. IFA was carried out with CHPV antisera as described [11]. When ≥ 50% infected wells showed plaque formation/IFA positive, presence of CHPV was confirmed.

#### (ii) *In ovo *and *in vivo *systems

Following inoculation with different dilutions of the virus, mortality was recorded 48 hr PI in embryonated eggs and infant mice. Mortality among ≥ 50% of the infected eggs/mice was considered indicator of the CHPV positivity.

#### (iii) Nested PCR

For cDNA synthesis 5 μl of RNA was added to a reaction mix containing 0.5 μl RNasin (Promega, Madison, USA 40 unit/μl), 1 μl of 10 μM CHPNDGF3 forward primer and incubated at 65°C for 5 min. A mixture containing 4 μl DNase-RNase free water, 4 μl of 5× AMV RT buffer, 1 μl of 25 mM dNTP' mix (Promega, Madison, USA), 0.5 μl RNasin, 1 μl AMV Reverse transcriptase (Promega, Madison, USA 10 unit/μl) was added to above tube and incubated at 42°C for 1 hr. 20 μl of cDNA were mixed with 5 μl each of CHPNDGF3 and CHPNDGR3 primers (10 μM), 1 μl of dNTPs (25 mM, Promega, Madison, USA), 10 μl of 10× PCR buffer, 10 μl of MgCl_2 _(25 mM), 1 μl (5 units) of AmpliTaq Gold (Applied Biosystems International, Foster City, CA) and DNase-RNase free water to make the volume of 100 μl. The first PCR was done with denaturation at 94°C for one min, annealing at 45°C for 30 sec. and extension at 72°C for 45 sec. For the second PCR, 10 μl of the product of first PCR was used as a template. The second PCR was performed with denaturation at 94°C for one min, annealing at 45°C for 30 sec. and extension at 72°C for 30 sec. The primers used were CHPNDGF4 and CHPNDGR4 in same quantity (Table 1). Both the PCRs were done for 35 cycles each. The PCR products were subjected to electrophoresis on 2% agarose gels. The expected size of the first PCR was 252 base pair and nested PCR product was 198 base pairs. Negative controls were included between samples and subjected to the entire PCR protocol. Pre-amplification and post-amplification were conducted on the different wings of the laboratory.

### Clinical Samples and Controls

Sensitivity and specificity of the real-time one step RT-PCR was evaluated using field collected serum samples. The samples included 42 sera from encephalitis cases from Warangal, an area endemic for CHPV, 10 Japanese encephalitis (JE) patients, eight encephalopathy patients associated with Chikungunya (CHIK) and 32 apparently healthy controls.

## Results

### Real-time one step RT-PCR Standardization

**Figure 2:**
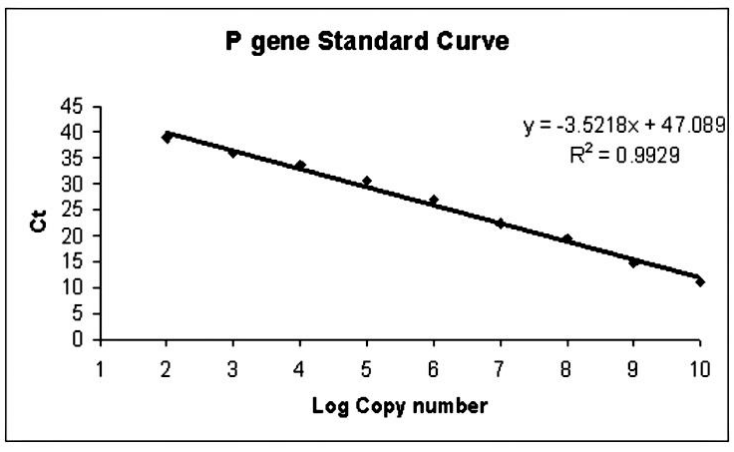
**Standard curve plot of log 10 diluted standard of P gene IVT RNA**. Log copy number were plotted against Ct. Plot represent mean of triplicate amplification of each dilution. The coefficient of determination (R^2^) and the equation of regression curve (y) were calculated.

Different concentration of primers and probe (100–600 nM) were used for the amplification reaction and Ct values were recorded. The 500 nM of probe and forward primer and 300 nM reverse primer were found optimal for real-time one step RT-PCR. Optimum concentrations of the primers and probe were used to assess the copy detection limits of RNA transcripts and dynamic range of real-time one step RT-PCR assay. The lower detection limit was approximately 100 copies of IVT RNA/reaction (Ct = 38.9). Strong linear correlation (R^2 ^> 0.99) was obtained between Ct values over a range from approximately 10^2 ^to 10^10 ^copies (Fig. 2) of IVT RNA/reaction.

### Reproducibility

Using replicate 10-fold serial dilutions of the IVT RNA, variability was evaluated for each dilution. Over the linear range of the assay, the coefficient of variation of the mean Ct values between runs was 5.91–1.18 (Table 2), indicating excellent reproducibility at high as well as low viral IVT RNA copies.

**Table 2:** Coefficient of variation (CV%) for the One Step real time RT-PCR assay.

(Copies of IVT RNA)	Ct	SD	CV%
10^10^	11.25	0.34	3.02
10^9^	14.71	0.87	**5.91**
10^8^	19.71	0.30	1.52
10^7^	23.14	0.54	2.33
10^6^	26.88	0.45	1.67
10^5^	30.39	0.46	1.51
10^4^	33.81	0.40	**1.18**
10^3^	35.80	0.56	1.56
10^2^	38.91	0.53	1.36

Standard Deviation (SD) and mean Ct vales were used for the calculation of CV%. Minimum and maximum vales as have shown in bold.

### Sensitivity of different systems

When the same sets of virus dilutions were used for infecting different host systems or PCR formats, real-time one step RT-PCR and nested RT-PCR scored to be most sensitive, both assays being able to detect CHPV RNA down to 1.2 × 10^0 ^PFU/ml (Table 3). Sensitivity of real-time RT-PCR and nested RT-PCR was found comparable, however, the first PCR of the nested PCR was 100 times less sensitive (Data not shown). Among the different hosts, lowest sensitivity was recorded for PS and Vero cells (down to 1.2 × 10^3^PFU/ml). Sand fly and RD cells as well as mice and embryonated eggs could detect the virus down to 1.2 × 10^2 ^PFU/ml.

**Table 3:** Comparison of different systems for the detection of CHPV.

Host/Method	PI (hours)	Limit of detection (≥) (Replicates +ve for CHPV/Total no of replicates)
RD cells	48	1.2 × 10^2 ^(2/4)
PS cells	48	1.2 × 10^3 ^(2/4)
Vero Cells	48	1.2 × 10^3 ^(3/4)
Sand fly Cell Line	48	1.2 × 10^2 ^(3/4)
Chick Embryo	48	1.2 × 10^2 ^(2/4)
Mice	48	1.2 × 10^2 ^(2/4)
Real Time One step RT-PCR	-	1.2 × 10^0 ^(3/3)
Nested RT-PCR	-	1.2 × 10^0 ^(3/3)

Limit of detection has been scored positive when ≥ 50% replicates showed CHPV by IFA, plaque formation/death or PCR amplification. Limit of detection has been presented in terms of PFU/ml.

### Sensitivity and specificity of real-time one step RT-PCR with clinical specimens

The clinical samples were assayed for CHPV RNA by real-time one step RT-PCR and nested RT-PCR. Fourteen serum samples (Warangal) were tested positive by both the methods with RNA copies ranging from 7.4 × 10^3^-1.3 × 10^5^copies/ml. Rest of the serum samples were found negative by both the methods. The sensitivity and specificity of the assay was 100% (Table 4) when nested RT-PCR was considered as the gold standard. No cross reactivity was detected with RNA extracted from JE and CHIK viruses as well as healthy controls.

**Table 4:** Comparison of nested RT-PCR with real-time one step RT-PCR for different samples.

Category	* Positive (by both nested RT-PCR and real-time one step RT-PCR)	* Negative (by both nested RT-PCR and real-time one step RT-PCR)	Total
CHPV suspected cases	14	28	42
CHIK virus associated encephalitis	0	11	11
JE cases	0	10	10
Healthy individuals	0	32	32

* No discrepancy was observed between the results of nested RT-PCR and real-time one step RT-PCR.

## Discussion and conclusion

Real-time RT-PCR has drawn significant attention in recent years as a molecular tool for detecting nucleic acids especially in virology due to rapidity, sensitivity, reproducibility and reduced risk of contamination [8,12,13]. It is also being used to quantitate viral load for studying the effect of antiviral agents both *in vitro *and in humans [14].

The present study reports development of a real-time one step RT-PCR assay for the quantitation of CHPV, which is highly sensitive (detection limit = 100 copies of IVT RNA/reaction.) and reproducible at both high and low IVT RNA copies. When the same sets of virus dilutions were used for infecting different host systems or PCR formats, real-time one step RT-PCR and nested RT-PCR found to be most sensitive. Among the host systems RD cells, sand fly cells, embryonated eggs and infant mice were most susceptible while Vero and PS cells were least susceptible to CHPV. Though RNA detection by both PCRs may not indicate infectious virus, both amplification techniques were 100-fold more sensitive than the most susceptible host systems. Susceptibility of different cells to viruses depends on several factors. The first step, i.e., the initial entry of the virus is determined by the availability and number specific receptors/co-receptors and attachment factors [15]. Subsequent to the entry, complex interactive mechanisms regulate replication, packaging and release from the infected cell. The differential sensitivity of different host systems may be the result of one or more of the above-mentioned factors. CHPV is known to replicate in a number of cell lines and living systems [16]. However, when clinical material positive for CHPV RNA is used for virus isolation, the success rate is significantly low indicating the need for a high virus load for replication. In an earlier study, real-time PCR was compared with culture technique for quantitative assessment of viral load in children naturally infected with respiratory syncytial virus [17].

We also assessed the diagnostic potential of real-time one step RT-PCR for CHPV. It may be noted that due to rapid progression of the disease, detection of viral RNA is the better diagnostic marker [2,3]. The real-time one step RT-PCR assay was found as sensitive as nested RT-PCR when compared using serial dilutions of the virus. However, in comparison with conventional RT-PCR, the fluorogenic assay presents many advantages, the most important being a closed system in which the tube is never opened post amplification, reducing the possibility of cross-contamination as well as higher validity of positive results due to the presence of two no template controls. In addition, this technique is rapid, allowing several samples to be processed simultaneously. This was clearly demonstrated when 42 encephalitis samples were processed individually by the two PCR assays, both detected the same samples (n = 14) as CHPV-RNA positive. Moreover, neither JE/CHIK virus infected encephalitis/encephalopathy patients' samples nor the 32 control samples (from healthy individuals) were reactive by both the methods. Thus, at cutoff Ct of 39.0, the sensitivity and the specificity of real-time assay was 100%. A cut off Ct of 42 was set for determining the specificity of real time PCR for Epstein-Barr virus (detection level = 100 copies/ml) [18]. However, Botero et al reported the superiority of nested PCR over real time PCR during a comparative study with cytomegalovirus in subgingival samples [19].

In conclusion, we observed that the newly developed real-time one-step RT-PCR assay is a valuable tool for the rapid detection and quantitation of CHPV. The technique may also find application during epidemic investigations in rapid diagnosis as well as a quantitative tool during antiviral and vaccine efficacy studies.

## Competing interests

The authors declare that they have no competing interests.

## Authors' contributions

SK has designed the experiments and standardized real-time one step RT-PCR. RSJ and ABS contributed in the animal/egg experiments and cell culture work respectively. BVT facilitated the clinical samples and helped in data analysis. ACM has reviewed the manuscript critically and coordinated the project. VAA has conceived the concept, participated in the design of the study and critical analysis of the data. All authors read and approved the final manuscript.

## Pre-publication history

The pre-publication history for this paper can be accessed here:


